# Novel Biomarkers of Renal Dysfunction and Congestion in Heart Failure

**DOI:** 10.3390/jpm12060898

**Published:** 2022-05-29

**Authors:** Agata Zdanowicz, Szymon Urban, Barbara Ponikowska, Gracjan Iwanek, Robert Zymliński, Piotr Ponikowski, Jan Biegus

**Affiliations:** 1 Institute of Heart Diseases, Medical University, 50-556 Wroclaw, Poland; agatazdanowicz@gmail.com (A.Z.); giwanek95@gmail.com (G.I.); robert.zymlinski@umw.edu.pl (R.Z.); piotrponikowski@4wsk.pl (P.P.); janbiegus@gmail.com (J.B.); 2 Student Scientific Organization, Institute of Heart Diseases, Medical University, 50-556 Wroclaw, Poland; barbara.ponikowska@student.umw.edu.pl

**Keywords:** heart failure, congestion, renal dysfunction, biomarker

## Abstract

Heart failure is a major public health problem and, despite the constantly emerging, new, effective treatments, it remains a leading cause of morbidity and mortality. Reliable tools for early diagnosis and risk stratification are crucial in the management of HF. This explains a growing interest in the development of new biomarkers related to various pathophysiological mechanisms of HF. In the course of this review, we focused on the markers of congestion and renal dysfunction in terms of their interference with cardiovascular homeostasis. Congestion is a hallmark feature of heart failure, contributing to symptoms, morbidity, and hospitalizations of patients with HF and has, therefore, become a therapeutic target in AHF. On the other hand, impaired renal function by altering the volume status contributes to the development and progression of HF and serves as a marker of an adverse clinical outcome. Early detection of congestion and an adequate assessment of renal status are essential for the prompt administration of patient-tailored therapy. This review provides an insight into recent advances in the field of HF biomarkers that could be potentially implemented in diagnosis and risk stratification of patients with HF.

## 1. Introduction

Heart failure (HF) is a multifaceted medical condition with a complex pathogenesis. Implementation of biomarkers that provide insight into a variety of biological processes involved in HF progression will potentially enable better diagnosis and prognosis of this disease [1]. Although many biomarkers have been identified, only a few have been incorporated into clinical practice, while others have yet to prove their applicability. In the course of this review, we explored the capabilities of the biomarkers related to renal dysfunction and congestion, which constitute the major pathological pathways of heart failure (HF).

Congestion is one of the most important mechanisms of heart failure (HF) development as well as being a therapeutic target during acute heart failure (AHF) episodes [1,2,3]. The pathophysiological processes underlying congestion in HF are multidirectional and go far beyond “simple” tissue fluid retention [2]. The HF-induced inability to maintain and control water-ion homeostasis leads to several disturbances that result in fluid accumulation and misdistribution [4,5]. Congestion is also the leading cause of hospitalizations for HF and is imminently associated with a poor outcome [6,7,8]. On the other hand, kidneys play a pivotal role in the management of the homeostasis and, therefore, significantly contribute to the development and reduction of fluid overload in HF. 

The incorporation of biomarkers that are involved in the aforementioned mechanisms might potentially increase the complexity of clinical assessment and improve the prognosis of HF patients by personalizing treatment for different phenotypes of the disease. The classification of selected biomarkers is presented in Table 1. 

## 2. Natriuretic Peptides 

Natriuretic peptides, particularly B-type natriuretic peptide (BNP) and its precursor N-terminal pro-B-type natriuretic peptide (NT-proBNP), have an established role as gold-standard biomarkers in HF management [9,10]. This view is supported by ESC guidelines, which advocate their application in HF diagnosis and prognosis [11]. NPs exert a wide range of biological effects with the primary aim to maintain blood pressure–volume homeostasis. These molecules are released in response to an elevated intracardiac pressure, which correlates with HF severity [9]. Furthermore, they contribute to the identification of new HF biomarkers and, since the discovery of benefits from neprilysin inhibition, they have become a novel therapeutic target [12]. Although their significance and clinical application in HF are undeniable, they possess certain limitations. Their lack of specificity has resulted in their tendency to increase in many medical conditions (such as arrhythmias, valvular heart disease, pulmonary hypertension, pulmonary thromboembolism, and sepsis) [13,14]. It has been demonstrated that their concentration might be altered by several confounding variables, including age, kidney function, and body mass index [14]. In some groups of patients, the use of NPs is unsatisfactory. They do not serve their diagnostic purpose in patients with renal disease at any stage. Additionally, their application in guiding decongestion therapy in patients on hemodialysis is not recommended [10]. Concerning their utility as markers of congestion, it should be pointed out that, since they are released in response to stretch and cardiac transmural pressure, they solely reflect intravascular volume overload and do not serve as a proxy for interstitial fluid accumulation. The biomarkers presented in the course of this review could, perhaps, complement NPs in these “gray areas” and provide additive clinical value in the HF setting.

## 3. Biomarkers of Renal Dysfunction and Injury

The relationship between the heart and renal dysfunction has been widely described and investigated. Mutual cardiac and renal dysfunction worsens the prognosis of patients with the so-called cardiorenal syndrome [15]. Classical serum creatinine level assessment can be misleading. The transient creatinine rise during decongestive therapy may represent the physiological response to fluid removal (hemoconcentration) and does not unequivocally reflect the renal injury or dysfunction [16]. Although many molecules have been postulated as potential biomarkers for the assessment of cardiorenal syndrome, only a few have been thoroughly evaluated. Notable among them are neutrophil gelatinase-associated lipocalin (NGAL), kidney injury molecule-1 (KIM-1), cystatin C (cysC), N-acetyl-ß-D-glucosaminidase (NAG), fibroblast growth factor 23 (FGF-23), and natriuresis. The summary of prognostic impact of selected biomarkers in HF is presented in Table 2, the predictive characteristics of the biomarkers are presented in Table 3.

### 3.1. NGAL

Neutrophil gelatinase-associated lipocalin is a siderophore molecule associated with neutrophils’ activation and an injury of epithelial cells, most interestingly, those located in the kidney. NGAL is also described as an iron-traffic regulator [12,48]. The molecule was originally proposed as an early marker of acute kidney injury (AKI), which anticipates the increase of serum creatinine [49].

#### 3.1.1. Clinical Value in Diagnosis and/or Prognosis

The role of NGAL in heart failure (HF) is multidimensional. It has been proven to be independently associated with poor prognosis in terms of mortality and rehospitalizations [20,21,22,50,51,52,53]. An elevation of NGAL has been described as a predictor of cardiorenal syndrome 1 in the population of patients admitted to the hospital due to HF [52,53]. Moreover, increased NGAL was observed in a population of HF patients that did not present symptoms of AKI (defined as elevated serum creatinine level) [22]. 

It is noteworthy that there is some evidence that NGAL expression is related to heart failure, possibly as a sign of neutrophil activation and inflammation [54]. Further reports on correlations between NGAL and inflammatory mediators, such as tumor necrosis factor alpha or matrix metallopeptidase [55], might provide additional information.

#### 3.1.2. Practical Considerations and Limitations

NGAL’s superiority over creatinine resides in its independence from diuretic therapy since it reflects renal injury rather than kidney function [56]. On the other hand, further studies, such as the Acute Kidney Injury N-gal Evaluation of Symptomatic Heart Failure Study (AKINESIS) and Placebo-Controlled Randomized Study of the Selective A(1) Adenosine Receptor Antagonist Rolofylline for Patients Hospitalized with Acute Decompensated Heart Failure and Volume Overload to Assess Treatment Effect on Congestion and Renal Function (PROTECT), have not confirmed its advantage over creatinine in the prediction of an adverse outcome or worsening renal function in acute heart failure patients [57,58,59].

### 3.2. KIM-1

Kidney injury molecule-1 is a transmembrane glycoprotein expressed in the proximal tubule [60]. KIM-1 is absent in the healthy tubule and its synthesis can be induced by ischemia, toxic injury, or the process of dedifferentiation of the epithelium [61,62,63]. It is also related to the conversion of the epithelial cell into the phagocyte [64]. KIM-1 has been thoroughly evaluated as an early marker of AKI.

#### 3.2.1. Clinical Value in Diagnosis and/or Prognosis

A recent meta-analysis of 14 studies and 3300 patients confirmed its value as a diagnostic tool for AKI, which reached the sensitivity of 0.74 and specificity of 0.84 [65]. KIM-1 has also been proposed as a tool for monitoring nephrotoxicity during pharmacotherapy [62,66]. Levels of KIM-1 in the symptomatic HF population have been reported to be higher than in healthy controls, regardless of the glomerular filtration status. Moreover, KIM-1 correlated with the New York Heart Association (NYHA) classification and left ventricular ejection fraction (LVEF) [67]. Different studies have shown its correlation with the n-terminal pro-brain natriuretic peptide (NT-pro-BNP) levels and increased risk of death and HF hospitalizations, independent of the initial glomerular filtration rate (GFR) [68,69]. KIM-1 was also evaluated as a prognostic factor of the all-cause mortality in chronic heart failure patients in a 10-year follow-up, but was not proven to be its independent predictor [18].

#### 3.2.2. Practical Considerations and Limitations

KIM-1, in comparison to the rest of the tubular injury markers (NGAL, NAG, CysC), has been most thoroughly evaluated as a drug-induced nephrotoxicity monitoring tool. Conversely, its role in the mortality prognosis of HF remains unclear and is a subject of many studies. 

### 3.3. CysC

Cystatin C is a protein produced by all nucleated cells. CysC is filtered, reabsorbed, and catabolized in the proximal tubule [70]. CysC is an alternative for the serum creatinine for the GFR calculation. The level of CysC is not affected by muscle mass or diet and depends on age, sex, and race less than creatinine. Conversely, it can be affected by smoking, inflammation, obesity, thyroid dysfunctions, glucocorticosteroids, and malignant processes [71,72]. Such characteristics prompted researchers to evaluate its value in the cardiorenal syndrome assessment. 

#### 3.3.1. Clinical Value in Diagnosis and/or Prognosis

The Heart and Soul study revealed that an increased level of CysC predicts all-cause mortality, cardiovascular events, and incidence of HF among ambulatory patients with coronary heart disease [73]. A meta-analysis, which included 10 prospective studies and 3155 patients, showed that an elevated CysC level is associated with an increased risk of all-cause mortality and rehospitalizations in the HF population, independently of creatinine and GFR [74]. CysC is reported to be increased in hypertensive heart failure with preserved ejection fraction (HFpEF) patients and, therefore, associated with left ventricular diastolic dysfunction and collagen alterations [75,76]. CysC is correlated with left ventricular diastolic diameter, left ventricular ejection fraction, and NYHA class [77]. Some studies suggest that CysC can predict in-hospital mortality [78] or 2-year cardiac events’ incidence better than NT-proBNP in AHF patients [77]. 

#### 3.3.2. Practical Considerations and Limitations

Although CysC can constitute a valuable complementary biomarker in the AKI diagnosis and prognosis assessment in heart failure patients, it does not provide much additional data compared to the other renal tubule injury markers. More sophisticated aspects of the cystatin C value in heart failure management require further studies. 

### 3.4. NAG 

N-acetyl-ß-D-glucosaminidase is the brush border enzyme expressed in proximal tubule cells [79]. As with the aforementioned biomarkers, such as NGAL, KIM-1, and CysC, it reflects the tubular damage and has been used as an early marker of AKI [67]. 

#### 3.4.1. Clinical Value in Diagnosis and/or Prognosis

An increased level of NAG in CHF patients in comparison to healthy controls has been noticed regardless of GFR. The NAG was also the predictor of death, heart transplantation, cardiovascular event, or HF hospitalization [68]. NAG was reported to be the strongest predictor among the novel renal biomarkers, NGAL and KIM-1, of 10-year all-cause mortality in HF patients [18]. Further studies confirmed the NAG increment in stable HF and revealed its associations with NT-proBNP, NYHA class, and LVEF [67]. NAG, conversely to the serum creatinine level, rose significantly after the withdrawal of diuretics during decongestion HF therapy and returned to the baseline after its re-initiation. Therefore, it can be considered as a renal marker of decongestion exhaustiveness [80]. Connotations between the NAG level and an accelerated progression of chronic kidney disease in HF patients has been described [81,82].

#### 3.4.2. Practical Considerations and Limitations

Among novel tubular injury markers, NAG seems to be the strongest predictor of long-term mortality in CHF patients [18]. 

NAG has many advantages, including high sensitivity and a simple method of quantification (easily reproducible spectrophotometric enzymatic assays). As for limitations, it should be emphasized that this marker is nonspecific. Increased levels of NAG have been observed in conditions other than AKI, such as hyperthyroidism and rheumatoid diseases. Additionally, endogenous urea and other neurotoxic substances alter NAG’s concentration [80,81].

### 3.5. FGF-23

Fibroblast growth factor-23 is a hormone dominantly secreted by osteocytes and osteoblasts in bones; however, it can also be produced by liver and heart muscle under stress [82,83]. FGF-23 plays a role in phosphate homeostasis by inducing its renal excretion. Further, FGF-23 downregulates the synthesis of vitamin D and parathormone [84]. A number of studies have shown the associations between elevated FGF-23 and left ventricular hypertrophy [83]. FGF-23 has been particularly linked to concentric hypertrophy–compensated cardiac hypertrophy without dilation [85]. This initiated a search for FGF-23 and heart failure associations. 

#### 3.5.1. Clinical Value in Diagnosis and/or Prognosis

FGF-23 was reported to be significantly higher in the HF versus healthy controls’ groups [86]. Initially, small-cohort studies suggested that FGF-23 was a strong predictor of outcome in the HF group, even stronger than classical predictors such as GFR, age, brain natriuretic peptide, diabetes, left ventricular mass index, or LVEF [87,88]. Then, some controversies about the differences of FGF-23 impact on prognosis between HFpEF and heart failure with reduced ejection fraction (HFrEF) arose. Several studies revealed that FGF-23 promotes HFpEF but predicts events in HFrEF [24,89]. Higher levels of FGF-23 were associated with volume overload, more frequent failure in reaching optimal doses of angiotensin-converting enzyme inhibitors/angiotensin II receptor blockers, and increased all-cause mortality and HF hospitalizations [23]. Conversely, another study showed that FGF-23 did not present an advantage over classical parameters in predicting the outcome in HF patients [90]. The predictive value, distinctions between the role of FGF-23 in HFpEF and HFrEF, and its involvement in cardiorenal cross-talk require further investigation.

#### 3.5.2. Practical Considerations and Limitations

FGF-23 provides novel insight into the previously not evaluated field of the heart–bone axis; however, its predictive value and the differences between its significance in HFpEF and HFrEF have to be elucidated before its implementation into clinical practice.

### 3.6. Urinary Sodium Excretion

European Society of Cardiology (ESC) guidelines from 2021 recommend navigating decongestive therapy based on the urine sodium excretion [44]. 

#### 3.6.1. Clinical Value in Diagnosis and/or Prognosis

Indeed, a number of studies have confirmed the prognostic and diagnostic value of spot urinary sodium assessment [91,92]. In the cohort of chronic heart failure patients, a decrease in urinary sodium excretion was an ominous sign of forthcoming decompensation and hospitalization due to acute HF [26]. Furthermore, an initial low urinary sodium level and a lack of its increment during diuretic therapy were associated with poor diuretic response, the elevation of NGAL and KIM-1, and increased 1-year all-cause mortality [25,93]. The prognostic value of the urinary sodium level in acute heart failure seems to be the most powerful during the first days of hospitalization, at the beginning of the decongestive therapy, with probably limited value at discharge [94,95]. 

#### 3.6.2. Practical Considerations and Limitations

Urine sodium level, at the time of writing this article, is probably the most trustworthy and widely applied biomarker of kidney functions in heart failure, excluding serum creatinine [91].

This review did not entirely cover the topic of cardiorenal cross-talk biomarkers. The role of TIMP2/IGFBP7 ratio assessment is noteworthy, as it is commercially available in the NephroCheck Test. Two multicenter studies confirmed its value in the AKI prediction, with an AUC of 0.8, superior to all existing biomarkers [12,96].

## 4. Exosomes and Non-Coding RNA 

Exosomes are small vesicles excreted by a variety of cells. They are composed of a double layer of the lipid membrane and contain protein, lipid, mRNA, long non-coding RNA, circular RNA, microRNA, DNA, and other molecules [97,98]. Initially, exosomes were perceived as an excretion material from the cell. The turning point of exosomes investigations was the study that showed that exosomes from B-lymphocytes play an antigen-presenting role [99,100]. Currently, exosomes are considered to be a means of information’s transmission between the cells. Importantly, exosomes are the carriers of non-coding RNA [101]. Exosomes are structures that protect vulnerable RNA from the harsh environment. They can stably exist in the human body fluids and plasma, which makes it a promising marker for monitoring the pathophysiological processes. The exosome and the non-coding RNA are inseparable concepts, as exosomes are the means of transport for the unstable RNA molecules. The Human Genome Project revealed that only 3% of human DNA is coding proteins. The remaining 97% was considered junk DNA. Further findings rejected this concept, associating the non-coding RNA with mainly regulatory functions [102]. Non-coding RNA can be divided according to the length of nucleic acid and its structure into smaller fractions. The most clinically and scientifically interesting of them are microRNA (miRNA), circular RNA (circRNA), and long non-coding RNA (lncRNA). The role of exosomes and RNA in HF pathophysiology is still underinvestigated. Exosomal miRNA has been proposed as a regulatory molecule in, e.g., cardiomyocyte hypertrophy [103,104,105], cardiac fibrosis [106], and myocardial angiogenesis [107]. Different subtypes of miRNA have been evaluated in terms of their applicability in the clinical setting. Elevated serum levels of miRNA successfully identified acute and chronic heart failure patients from healthy controls and were associated with natriuretic peptide levels, wide QRS, dilatation of the left ventricle and atrium [108,109], NYHA class [110], mortality [111], and echocardiographic parameters [112]. Little is known about the clinical utility of circRNA and lncRNA. Nevertheless, several studies suggested their important role in the regulation of pathophysiological pathways in HF, including cardiac hypertrophy, ischemic remodeling, inflammatory process, and the regulation of intracellular Ca^2+^ level [102,113]. 

## 5. Multimarker Panels and Clustering

Heart failure is an end stage of cardiovascular diseases and a consequence of a number of pathological pathways. Multimarker evaluation can provide significant information regarding the disease phenotype by including molecules of a different pathophysiological origin [70,114]. Currently, the American College of Cardiology (ACC)/American Heart Association (AHA) heart failure guidelines suggest that assessing troponins, soluble suppression of tumorigenicity-2, and galectin-3 could enhance the natriuretic peptides’ predictive value in the risk stratification [115]. One of the most important limitations of multimarker assessment is the arbitrary choice of the biomarkers. Conversely, in a recent sub-analysis of the Treatment of Preserved Cardiac Function Heart Failure with an Aldosterone Antagonist (TOPCAT) study, the authors implemented a machine learning model, which automatically selected significant molecules (out of 49 available) to predict the outcome [116]. Importantly, the authors implemented clustering techniques to analyze the heterogeneity of the biomarkers. Clustering is the unsupervised machine learning technique that divides a set of numerical variables into smaller groups based on their similarity. Clusters are composed of variables that are consistent with each other but not with other clusters [116]. In the recent study, 577 urine peptides from heart failure patients and matched controls were analyzed [117]. Algorithm-based clustering automatically divided participants based on their urinary peptides profiles; 83% of non-HF controls were allocated into cluster 1, while 65% of HF patients were in cluster 2. This experiment revealed the natural, machine-detectable differences between HF and non-HF patients’ urinary profiles. Moreover, the study showed clustering techniques’ ability to automatically reveal the group of proteins that can present the diagnostic and therapeutic potential. 

## 6. Congestions 

### 6.1. Cancer Antigen 125 (CA-125) 

Cancer antigen 125 (CA-125), otherwise known as mucin 16, is a membrane glycoprotein synthesized by mesothelial cells. Traditionally, its use has been restricted to screening and therapeutic monitoring of ovarian cancer [118,119]. However, the CA-125 level has been found to be upregulated in other nonmalignant processes, such as heart failure. A growing body of evidence has implicated CA-125 in the pathophysiological processes underlying HF [120]. In the latter context, the glycoprotein is released in response to extravascular congestion and cytokine-induced inflammation as well as myocardial stress and injury [121]. Taking all this into consideration, CA-125, as a surrogate for fluid overload and inflammation in HF patients, emerges as a potential novel biomarker of this pathology [118,119]. 

#### 6.1.1. Clinical Value in Diagnosis and/or Prognosis

Several studies have established an association between the CA-125 concentration and clinical manifestation of congestion (including peripheral edema and serosal effusion) in the HF population [27,28,29,30,31,32,33,34,122,123,124,125,126,127,128,129]. The elevation of CA-125 is positively correlated with the severity of an extravascular congestion and is consistent with clinical, hemodynamic, and echocardiographic parameters of fluid overload [27,124].

Patients with more severe congestion had, on average, higher level of CA-125 (mean difference of 54.8 U/mL) than patients without congestion [31]. Among patients presenting to an emergency department with an acute dyspnea of uncertain origin, the highest CA-125 values were recorded in patients with AHF, particularly in those with a worsening heart failure phenotype [125].

Recently, there has been a substantial increase in the number of studies investigating a prognostic value of CA-125 in risk stratification of the HF population. Results of the various trials demonstrated that the elevation of the CA-125 level was positively correlated with an increased risk of mortality and readmission for HF. CA-125 remained a significant prognostic predictor of poor prognosis, even after adjustment for the relevant baseline covariates [28,29,30,32,33,34,126,127,128]. CA-125 had a similar positive predictive value as NTproBNP in AHF but was superior to NTproBNP when effusion was present [129]. Key findings from a recent sub-analysis of BIOSTAT-CHF are in line with these findings. It was reported that CA125 was positively correlated with the clinical parameters of congestion (namely, peripheral edema, hepatomegaly, orthopnea, etc.), and a higher level of plasma CA125 was associated with an increased risk of a 1-year all-cause mortality and the composite of death/HF readmission [123]. In the group of AHF patients, those who had a high CA-125 level and low concentration of NT-proBNP at admission had significantly worse prognoses than patients with low CA-125 levels and low NT-proBNP. Likewise, those with high NT-proBNP and high CA125 levels had the least favorable prognoses [30]. Thus, it can be inferred that the implementation of CA125 in conjunction with NT-proBNP is more accurate in risk stratification of patients with AHF than the conventional use of NT-proBNP alone. 

#### 6.1.2. Role of CA-125 in Heart Failure Treatment 

The substantial number of studies have recently demonstrated that the concentration of CA-125 changes with the degree of fluid overload and, thus, may reflect treatment-induced alterations in a patient’s volume status [31,32,130,131,132]. Considering this finding, CA125 emerges as a potential tool to guide effective decongestion therapy. The authors of the CHANCE-HF trial concluded that CA-125-tailored diuretic therapy was superior to a conventional one in reducing the risk of the composite of 1-year death or AHF readmission [27]. CA-125-guided diuretic therapy was associated with a significant reduction in death and readmission for AHF at 30 days. The CA-125-guided group exhibited a considerably greater improvement in renal function (increase in GFR) in comparison to the standard treatment group [133]. Given the long half-life (over a week) of CA-125, it does not provide any information about acute response to therapy [132]. It might, however, be a reasonable approach to measure CA-125 concentrations at admission for HF as well as at least 7 days after the initial examination. 

#### 6.1.3. Practical Considerations and Limitations

CA-125 is associated with congestion, right-sided HF parameters, and an increased risk of adverse clinical events in AHF, beyond standard prognostic factors such as natriuretic peptides [133]. Broad availability, standardized measurement, and low cost make it a compelling candidate for routine use in decompensated HF. Variations in CA-125 concentrations according to the clinical situation make it a potential tool for both monitoring and guiding HF treatment following a decompensated HF event [133]. There are several limitations that currently prevent CA-125 from being used in the HF setting. These include an insufficient understanding of CA-125 biology, a lack of an optimal cutoff value, and indefinitive data from large multicenter trials [133]. Hence, future large-scale research is required to validate the role of CA-125 in heart failure. 

## 7. Neurohumoral Activation

Neurohormonal activation (expressed by stimulation of the sympathetic system and RAAS) is believed to be a central mechanism underlying HF pathophysiology. Reduced cardiac output induced by myocardial dysfunction activates the neurohormonal axis to maintain hemodynamic stability. Although activation of this system is presumed to be compensatory, over the long term, it contributes to further deterioration of cardiac systolic function and development of heart failure symptoms [134,135]. While a substantial body of evidence points to a prognostic role of neurohumoral markers, they are not measured in daily practice. It stems from the fact that the treatment of HF is based on the use of angiotensin-converting enzyme inhibitors and beta-adrenergic receptor blockers, which can significantly modulate plasma concentrations of these hormones, thereby interfering with their interpretation and reducing their predictive value in risk stratification [135]. Therefore, further research is required to identify a stable, yet sensitive, and relatively easy to measure biomarker. 

### 7.1. Adrenomedullin

Adrenomedullin (ADM) is a newly discovered vasoactive and natriuretic peptide hormone involved in cardiorenal regulation. ADM is widely distributed in the cardiovascular system, kidneys, and adrenal glands [135,136,137]. This hormone is released by endothelial and vascular smooth muscles in response to fluid overload and endothelial barrier disruption. ADM-induced preservation of vascular integrity contributes to the reduction in tissue congestion. Conversely, the inhibition of the ADM results in vascular leakage and systemic/pulmonary edema development [137,138]. Hence, elevated levels of ADM are frequently observed among patients with HF or septic shock. This finding stems from the fact that vascular leakage and organ hypoperfusion are commonly encountered in these two pathologies [138,139]. It stimulates vasodilatation, positive inotropic action, and cardiac hypertrophy inhibition. Thus, it decreases blood pressure and increases blood flow [140,141,142,143]. It was observed that even relatively low doses of ADM produced significant vascular dilatation. This finding suggests that, in conditions such as HF, ADM might be in the range that is capable of affecting vascular tone [143,144]. Additionally, by suppressing RAAS, ADM exerts diuretic and natriuretic effects. Its action contributes to an improvement in the glomerular filtration rate [145,146,147]. Given the cardioprotective role of ADM, it has been suggested that it has a potential to become a therapeutic target in patients with HF [148].

#### 7.1.1. Clinical Value in Diagnosis and/or Prognosis

ADM might be utilized as a marker of congestion in patients with a new onset and worsening HF [35,36,149,150,151,152,153] It has been observed that patients with a high concentration of this vasoactive peptide exhibited more severe signs of congestion (edema, orthopnea, and elevated jugular venous pressure) [36]. The association between a degree of fluid overload and bio-ADM concentration at baseline remained significant even after adjustment for a multivariable model [35]. A strong association between the ADM level and the degree of hemodynamic instability, impaired cardiac function, and prognosis in cariogenic shock was demonstrated in a recent cohort study. Results revealed that a high level of ADM (observed in a non-survivor group) was positively correlated with an impaired cardiac index, mean arterial pressure, and central venous pressure [35]. MR-proADM was superior to BNP and troponin for predicting 90-day all-cause mortality in AHF patients with acute dyspnea [149]. Findings of the PROTECT trial indicated that, among patients hospitalized for AHF, elevation of bio-ADM at discharge reflected residual congestion and was related to an increased risk of early rehospitalization [153]. When combined with BNP and NT-proBNP, Mr-proADM provided significant incremental predictive value for 90-day mortality [150].

#### 7.1.2. Practical Considerations and Limitations

The use of MR-proADM in the diagnosis of AHF is limited due to its low specificity. MR-proADM is elevated in various medical conditions (systemic hypertension, myocardial infarction, kidney failure, or sepsis) [151]. It is also crucial to point out that the major disadvantage of MR-proADM is the fact that its level is influenced by a patient’s baseline covariates. Higher concentrations of MR-proADM were reported in female elderly patients, kidney disease, and systolic dysfunction. The biomarker’s level decreased with a higher body mass index [152]. Further large, multi-center studies are required to establish the role of this biomarker and determine its value in conjunction with other biomarkers. 

### 7.2. Arginine Vasopressin and Copeptin

Arginine vasopressin (AVP) is an antidiuretic and vasoconstrictive peptide hormone, released in response to hyperosmolality and hypovolemia. It plays a crucial role in hemodynamics and osmoregulation [154].

Key functions of AVP include solute-free water reabsorption in kidney tubules, an increase in peripheral vascular resistance, and a consequential rise in arterial blood pressure [155,156]. Moreover, AVP is a crucial component of the endocrine stress response, triggering ACTH and cortisol release [154]. Due to the poor stability and short half-life (15–20 min) of AVP, copeptin (CT-proAVP, C-terminal segment of pre-provasopressin) was introduced into clinical practice as a reliable surrogate of this hormone [154,155].

#### 7.2.1. Clinical Value in Diagnosis and/or Prognosis

The evidence of AVP elevation in HF patients has been well documented in the literature [157,158,159,160]. Among patients with HFpEF, AVP was independently associated with LV hypertrophy and a higher risk of death or HF readmissions. In line with the previous findings, copeptin has been found to be a strong predictor of poor prognosis in the HF population [37]. The BACH study indicated that patients with elevated copeptin levels (especially those with hyponatremia) had significantly higher 90-day mortality and a higher risk of rehospitalization [38]. Furthermore, a meta-analysis, which included 10 prospective cohort studies, revealed that an increased concentration of copeptin was positively correlated with an all-cause mortality in the HF population. The prognostic role of copeptin was found to be equivalent to NT-proBNP for all-cause mortality in patients with HF [38]. Copeptin provided independent prognostic information in severe HF, although its prognostic impact was inferior to NT-proBNP [161].

#### 7.2.2. Practical Considerations and Limitations

Although the biological effect of this biomarker has been widely explored, the optimal use of copeptin in the HF setting remains a question of debate. Copeptin is a sensitive and easily measurable surrogate of AVP, yet it is more stable and has a longer half-life. CT-proAVP exhibits low specificity. The elevation of AVP/CT-proAVP has been associated with a number of diseases, particularly those that are characterized by acute stress [162]. The proper assessment of copeptin’s results requires knowledge about confounding factors that interfere with its concentration. Copeptin has been shown to be increased in males and to be associated with a decreased glomerular filtration rate, probably as a result of decreased renal copeptin clearance [154,155]. This marker is noteworthy for its potential value in identifying high-risk patients in a critical condition and, thus, implementing individualized patient care.

### 7.3. Chromogranin A

Widely recognized as a major marker of neuroendocrine tumor (NET), chromogranin A (CgA) is an acidic protein and a pro-hormone of an active particle, which potentially exerts a biological effect in CHF [163,164]

#### 7.3.1. Clinical Value in Diagnosis and/or Prognosis

A considerable interest in this protein stems from the fact that CgA has been found to be related to the clinical deterioration and higher risk of mortality in patients with AHF and CHF [39,165,166]. The concentration of CgA was measured in a group of 160 patients with CHF to evaluate the association between the CgA level and HF severity (based on the NYHA scale). The results demonstrated that class IV NYHA patients had the highest level of CgA (median 545.0 ng·mL^−1^) while class I had the lowest concentration of this protein (median 109.7 ng·mL^−1^). Furthermore, it was concluded that CgA might be a predictive factor for mortality in HF [17]. CgA was found to have comparable prognostic value to that of NT-proBNP in AHF patients [163]. High concentrations of CgA were independently associated with 1-year death and hospitalization for heart failure [163].

#### 7.3.2. Practical Considerations and Limitations

Data regarding CgA measurement in cardiology are limited. Its role has been investigated only in studies with a small number of patients. In the current state of research, CgA plasma measurement as a biomarker in heart failure is still being explored and cannot be recommended for general use [163]. Furthermore, complex and extensive CgA processing has hampered its use as a routine biomarker due to methodological problems with its measurement [165].

## 8. Conclusions

With a growing prevalence and incidence of HF, novel and reliable diagnostic tools are thoroughly investigated. Currently, B-type natriuretic peptide (BNP) and N-terminal proBNP are the most recognized biomarkers for the diagnosis and treatment of HF; however, they do have certain limitations. They do not serve their prognostic role in some group of patients such as in those with renal failure and are not specific for HFpEF patients. Therefore, further research to explore new markers that would provide additional prognostic information, especially in areas uncovered by classic biomarkers, is required. Novel biomarkers certainly comprise those related to congestion and renal dysfunction, as, in fact, these pathological pathways are closely related to the development and progression of HF. Although it is important to note that the individual clinical value of the aforementioned biomarkers in diagnosis and prognosis is limited due to their lack of specificity, as such, the future of biomarker application in HF management relies on a multimarker panel and clustering strategies that would include a more specific combination of biomarkers representing different pathophysiological processes underlying HF. Multimarker panels’ analysis represents the great potential in prediction, risk stratification, and therapy tailoring in cardiovascular disease. A combination of various biomarkers reflects the cross talk of different pathophysiological pathways, including cardiac remodeling, inflammatory process, renal dysfunction, and neurohormonal activation, all of which play a significant role in heart failure. Machine learning techniques, such as clustering, can constitute helpful tools for elucidating the heterogeneity of the heart failure population and distinguishing important parameters from the immense amounts of data.

## Figures and Tables

**Table 1:** Classification of selected biomarkers based on pathophysiological pathways of heart failure.

Pathophysiological Pathway	Biomarkers
Kidney injury and dysfunction	Neutrophil gelatinase-associated lipocalin (NGAL) Kidney injury molecule-1 (KIM-1) Cystatin C (cysC) N-acetyl-ß-D-glucosaminidase (NAG) Fibroblast growth factor 23 (FGF-23) Natriuresis
Congestion	Cancer antigen 125 (Ca-125) Adrenomedullin (ADM) NT-proBNP
Neurohumoral activation	Adrenomedullin (ADM) Arginine vasopressin (AVP) Copeptin (CT-proAVP) Chromogranin A (CgA)
Wide spectrum of pathological pathways	microRNA

**Table 2:** Prognostic impact of selected biomarkers in heart failure.

Biomarker	Pathophysiological Mechanism	Clinical Applicability in AHF	Clinical Applicability in CHF
NAG	Tubulointerstitial damage	Worse clinical outcome (death, worsening HF) [17].	Increase in mortality and rehospitalization [18].
KIM-1	Tubulointerstitial damage	No impact on prognosis [19]. Correlation with worsening of renal function in AHF [20].	Increase in 10-year all-cause mortality [18]. Increase in mortality and rehospitalization [18].
NGAL	Tubulointerstitial damage	Strong prognostic indicator of 30-day outcome [21]. Correlation with worsening of renal function in AHF [20].	Increase in all-cause mortality and rehospitalization [22].
FGF-23	Renal function mineral metabolism	Increased risk of all-cause mortality and HF hospitalization [23].	Increased risk of mortality in HFrEF [24].
Spot urine sodium	Renal function	Low urinary sodium at hospital admission is independently associated with all-cause mortality [25].	Chronically low urine, high risk of hospitalization for decompensation [26].
CA-125	Congestion	Increase in mortality and readmission [27,28,29,30,31].	Increase in mortality and readmission [32,33,34].
ADM	Residual congestion neurohumoral activation	Increased risk of all-cause mortality and HF hospitalization [35]. High risk of early hospital readmission [36].	Increased risk of all-cause mortality and HF hospitalization [35].
AVP/CT-proAVP	Neurohumoral activation	Increase in 90-day mortality. High risk of rehospitalization [37]. Increase in all-cause mortality [38].	Increase in all-cause mortality [38].
Chromogranin A	Neurohumoral activation	Increase in mortality [39].	Increase in mortality [17].
MicroRNA	Broad spectrum of mechanisms and correlations with HF prognosis, depending on the specific molecule.		

**Table 3:** Predictive characteristics of selected biomarkers in heart failure.

Biomarker	Cutoff Value	Specificity	Sensitivity	AUC	Clinical Value
NGAL	84 ng/mL	0.6	0.8	0.72	Mortality in CHF [40].
NAG	4.69	-	-	0.708	AKI prediction in critically ill patients [41].
KIM1	1.62	0.44	0.80	0.757	AKI in ADHF [42].
FGF-23	1180 RU/mL	0.8	0.5	0.686	28-day mortality in cardiogenic shock [43].
Spot urine sodium	50–70 mEq/L	-	-	-	Diuretic response prognosis and evaluation [44].
CA-125	32 U/mL	0.72	0.83	0.784	1-year death in CHF [45].
MR-proADM	4.6 nmol/L	0.810	0,577	0.729	Myocardial injury [46].
	3.5	0.605	0.80	0.730	Mortality at 28 days [46].
CT-proAVP	112.5 pg/mL	87%	86%	0.91	Early diagnosis of acute myocardial infarction [47].
Chromogranin A	158 pmol/L	-	-	0.697	1-year death and hospitalization in AHF [17].

Data presented in the table are considered provisional. These biomarkers are a subject of ongoing studies and official guidelines are yet to be established.

## Data Availability

Not applicable.
